# Periodic Leg Movements during Sleep Associated with REM Sleep Behavior Disorder: A Machine Learning Study

**DOI:** 10.3390/diagnostics14040363

**Published:** 2024-02-07

**Authors:** Maria Salsone, Basilio Vescio, Andrea Quattrone, Sara Marelli, Alessandra Castelnuovo, Francesca Casoni, Aldo Quattrone, Luigi Ferini-Strambi

**Affiliations:** 1 Institute of Molecular Bioimaging and Physiology, National Research Council, 20054 Segrate, Italy; 2 Sleep Disorders Center, Division of Neuroscience, San Raffaele Scientific Institute, 20132 Milan, Italy; marelli.sara@hsr.it (S.M.); casoni.francesca@hsr.it (F.C.); ferinistrambi.luigi@hsr.it (L.F.-S.); 3 Neuroimaging Research Unit, Institute of Molecular Bioimaging and Physiology (IBFM), National Research Council (CNR), 88100 Catanzaro, Italy; b.vescio@unicz.it; 4 Biotecnomed S.C.aR.L., c/o Magna Graecia University, G Building, lev.1, 88100 Catanzaro, Italy; 5 Institute of Neurology, Magna Graecia University, 88100 Catanzaro, Italy; an.quattrone@hotmail.it; 6 Sleep Disorders Center, Division of Neuroscience, Vita-Salute San Raffaele University, 20132 Milan, Italy; castelnuovo.alessandra@hsr.it; 7 Neuroscience Research Center, Magna Graecia University, 88100 Catanzaro, Italy

**Keywords:** REM sleep behavior disorder (RBD), periodic leg movements during sleep (PLMS), Artificial Intelligence (AI), Machine Learning (ML)

## Abstract

Most patients with idiopathic REM sleep behavior disorder (iRBD) present peculiar repetitive leg jerks during sleep in their clinical spectrum, called periodic leg movements (PLMS). The clinical differentiation of iRBD patients with and without PLMS is challenging, without polysomnographic confirmation. The aim of this study is to develop a new Machine Learning (ML) approach to distinguish between iRBD phenotypes. Heart rate variability (HRV) data were acquired from forty-two consecutive iRBD patients (23 with PLMS and 19 without PLMS). All participants underwent video-polysomnography to confirm the clinical diagnosis. ML models based on Logistic Regression (LR), Support Vector Machine (SVM), Random Forest (RF), and eXtreme Gradient Boosting (XGBoost) were trained on HRV data, and classification performances were assessed using Leave-One-Out cross-validation. No significant clinical differences emerged between the two groups. The RF model showed the best performance in differentiating between iRBD phenotypes with excellent accuracy (86%), sensitivity (96%), and specificity (74%); SVM and XGBoost had good accuracy (81% and 78%, respectively), sensitivity (83% for both), and specificity (79% and 72%, respectively). In contrast, LR had low performances (accuracy 71%). Our results demonstrate that ML algorithms accurately differentiate iRBD patients from those without PLMS, encouraging the use of Artificial Intelligence to support the diagnosis of clinically indistinguishable iRBD phenotypes.

## 1. Introduction

The loss of muscle atonia and abnormal behaviors, such as sleep-related vocalization and/or complex motor behaviors, during the rapid eye movement (REM) sleep phase, characterize the REM sleep behavior disorder (RBD) [1,2]. The scientific interest in RBD is that its idiopathic (or isolated) form (iRBD) is now considered a neuropathological precursor of synucleinopathies like Parkinson’s disease (PD), Dementia with Lewy bodies (DLB), and Multiple System Atrophy (MSA) [3]. Data from a multicentric study revealed that the rate of phenoconversion to overt neurodegenerative disorder significantly increases in the presence of specific biomarkers including the olfactory deficit, mild cognitive impairment, erectile dysfunction, motor symptoms, abnormal DAT scan, color vision abnormalities constipation, REM atonia loss, and age [4].

Interestingly, about 70% of patients with RBD exhibit periodic leg movements during sleep (PLMS) at all sleep stages, especially during REM sleep [5,6,7]. PLMS are clinically defined as stereotyped and recurring movements of the lower limbs, such as extension of the big toe, and partial flexion of the ankle, knee, and hip [6,7,8]. However, these peculiar movement disorders are not exclusive to RBD. Indeed, they are symptoms belonging to the clinical picture of several sleep disorders, such as narcolepsy and obstructive sleep apnea, and are significantly associated with restless leg syndrome (RLS). On the other hand, distinct electrophysiological features including distribution across the night, periodicity, duration, amplitude, and time structure characterize PLMS in sleep disorders, thus suggesting a disease-specific phenomenon [9]. Finally, PLMS were also frequently reported in apparently normal older adults [8]. Therefore, the question that remains unsolved until now is whether the presence of PLMS in iRBD identifies a clinical subtype with a higher risk of phenoconversion. In this context, differentiating iRBD patients from those without PLMS could be of clinical relevance for future neurodegenerative risk.

Although the clinical history is accurate, PLMS are often misdiagnosed. The gold standard for a correct diagnosis is polysomnography (PSG). A PLMS index on PSG recording, calculated as the number of periodic leg movements per hour of sleep equal to or greater than 15 events/h, allows for the detection and quantification of PLMS [10]. The literature regarding the development of alternative methods for the automatic detection, scoring, and analysis of PLMS, as well as for differential diagnosis among iRBD phenotypes, is poor. In recent years, some Artificial Intelligence (AI) tools including Deep Learning (DL) and Machine Learning (ML) have been developed with good results. A new Deep Learning model (deepPLM) based on a single-lead electrocardiogram (ECG) signal from polysomnographic recordings was used for the automatic detection of PLMS in 52 subjects (26 controls and 26 patients) with good performances [11]. An AI-enabled method for the automatic classification of sleep disorders, including PLMS using ECG alone, has also been proposed [12]. ML algorithms such as the Random Forest (RF) model and Extreme Gradient Boosting (XGBoost) have been widely developed and successfully applied for the correct differential diagnosis of neurological diseases. Recently, it has been demonstrated that the RF model had excellent accuracy (94%), sensitivity (95%), and specificity (92%) in distinguishing iRBD subjects from healthy subjects [13]. In the current study, we aimed to evaluate the performance of ML algorithms (Logistic Regression (LR), Supporter Vector Machine (SVM) XGBoost, and RF) in differentiating iRBD patients with and without PLMS. Furthermore, we tested the hypothesis that the phenotype might be different between the two iRBD groups, with a different risk profile for future neurodegeneration.

## 2. Materials and Methods

### 2.1. Participants 

Forty-two patients who fulfilled the diagnostic criteria for iRBD were enrolled in this study. All subjects were thoroughly examined by neurologists to exclude cases in which other neurological diseases were present. A video-PSG was needed for the confirmation or exclusion of the presence of clinical/subclinical RBD and to detect the presence of PLMS. Significant muscle activity during REM sleep associated with abnormal movements and other behaviors detected in PSG recordings was required for the confirmation of the clinical or subclinical diagnosis of RBD [14], as required by the International Classification of Sleep Disorders—Third Edition (ICSD-3) criteria. Automatic labeling was used for scoring the sleep stages in the PSG recordings. Scored sleep stages were then checked manually by an expert sleep examiner in order to correct possible wrong scores. Regarding PLMS diagnosis, PLMS were scored according to the previously published criteria of the American Academy of Sleep Medicine [15] and were carefully differentiated from the phasic EMG activity on the anterior tibialis based on their regular periodicity [16]. A PLMS index value ≥ 15 events/h (abnormal value) [8,17] was used to divide our cohort into two iRBD subgroups: (i) iRBD with PLMS (iRBD-PLMS, value ≥ 15); (ii) iRBD without PLMS (iRBD, value < 15). To better characterize these two iRBD phenotypes, the presence of phenoconversion biomarkers has also been investigated, including (i) olfactory deficits/hyposmia; (ii) deficits in cognitive domains; (iii) cerebral hypometabolism showed on FDG-PET imaging; (iv) motor symptoms. In detail, olfactory deficits/hyposmia were considered when referred by the patients. Neuropsychological evaluation was performed by a specialist (SM) using the following criteria: Mini-Mental State Examination (MMSE) [18], Digit Span forward and backward [19]; Corsi block-tapping Test [20]; Rey’s List: learning, recall, and recognition [21]; Raven’s Progressive Matrices [22]; Attentive Matrices [22]; Verbal Fluency with Phonemic and Semantic cues [23]; Token Test [24]; and Copy of Rey–Osterrieth complex figure [25]. The presence of hypometabolism on FDG-PET imaging was evaluated on the basis of a previous neuroimaging visual investigation made by the patient. Finally, the Unified Parkinson’s Disease Rating Scale motor score (UPDRS-ME) [26] was used to evaluate the presence and severity of extrapyramidal signs. The exclusion criteria were as follows: (i) the diagnosis of other sleep disorders including RLS, obstructive sleep apnea syndrome (OSA), and narcolepsy; (ii) ongoing treatment with medications modifying REM sleep architecture and muscle tone, such as serotonin reuptake inhibitors. Before being included in the study, all participants provided their written informed consent. The experimental procedure was previously approved by the local Ethical Committee of the “Vita-Salute” San Raffaele University, Milan, Italy (no. 38, 8 March 2022).

### 2.2. Heart Rate Variability (HRV) Analysis

All study participants underwent a circadian HRV analysis to detect potential cardiac autonomic dysfunctions. HRV components during 24 h and related cardiac autonomic indices were calculated according to our previously published protocol [13,27]. 

### 2.3. Machine Learning Models

The discriminating performances of single HRV features were evaluated using a Receiver Operator characteristic (ROC) analysis. Logistic Regression (LR), Support Vector Machine (SVM) with linear kernel, Random Forest (RF), and eXtreme Gradient Boosting (XGBoost) ML models were trained on HRV features [13]. Leave-One-Out Cross-Validation (LOO-CV) was used during the training process to assess the training classification performances in discriminating iRBD with PLMS patients from iRBD without PLMS patients. Feature importances were computed and ML models were built using forward feature selection, starting from the feature with the highest importance and iteratively adding new features in descending order of importance. Each model was trained using LOO-CV, and the optimal model, with the optimal number of features, was chosen as the model with the highest classification accuracy. During the training phase, a grid search strategy was used to tune the hyperparameters of the ML models and maximize the performance.

### 2.4. Statistical Analysis 

All categorical variables (sex, RBD symptoms, and phenoconversion biomarkers) were compared using the Χ^2^ test. Continuous variables were compared using either Student’s *t*-test or Mann–Whitney U test after assessing distribution properties using the Shapiro–Wilk test.

ECG processing, extraction of R peaks, and preprocessing of RR intervals were performed using SciPy (version 1.8.0), Nolds (version 0.5.2), hrv-analysis (version 1.0.4), and BiospPy (version 0.8.0) libraries in the Python programming language (version 3.9.2). HRV features were computed using the PhysioNet HRV Toolkit [28] written in the C programming language. ML and statistical analysis were performed with the help of the caret R package (version 4.0.0) [29] and the R programming environment [30] (version 4.0.4, 2021, The R Foundation for Statistical Computing, Vienna, Austria).

## 3. Results 

The demographic, clinical, and neuropsychological features of the iRBD patients with and without PLMS are summarized in Table 1. No significant differences were found between the two groups in terms of age and sex distribution (Table 1). The iRBD-PLMS patients were slightly older and had a higher age at the onset of RBD symptoms than those with iRBD. The PSG evaluation revealed a statistically insignificant higher percentage of patients with PSG features without clinical signs in the iRBD-PLMS group. The education level was similar, although it was lower than that of iRBD in iRBD-PLMS patients. No significant differences were observed between the two RBD groups in terms of the neuropsychological battery performance (Table 1). A slight reduction within the normal range was detected in both groups regarding the attentive function. Finally, a similar distribution of phenoconversion biomarkers to synucleinopathies was detected in both groups (Table 2). Hyposmia and cognitive deficits were more frequently biomarkers reported in iRBD patients with and without PLMS (Table 2). Interestingly, the iRBD-PLMS group showed a significantly higher percentage of patients with at least one biomarker (Table 2). 

### 3.1. HRV Analysis

**Figure 1:**
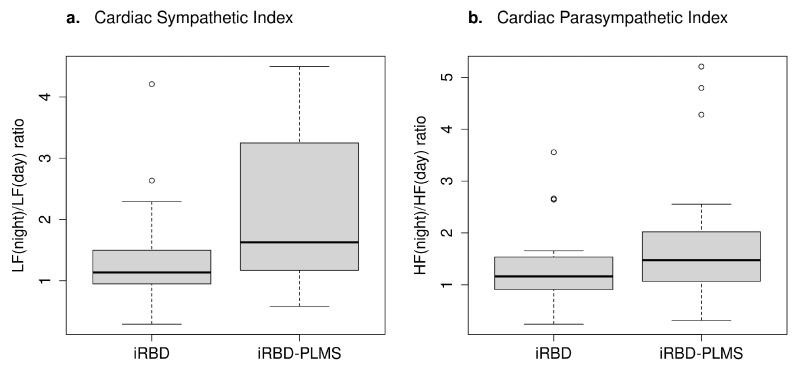
Box plots of (**a**) cardiac sympathetic (*p* = 0.38) and (**b**) cardiac parasympathetic (*p* = 0.19) index in iRBD patients with and without PLMS (distributions have been compared using Wilcoxon’s rank sum test). Please refer to [13] for the interpretation of the box plots and the definitions of the LF and HF power bands.

In 24 h recordings, we observed that autonomic indices increased in iRBD patients with PLMS compared to those without PLMS (Table 1). However, this increase was not statistically significant, and the previously published cut-off levels [13,27] did not show good classification performances, as individual values in iRBD patients with and without PLMS overlapped (Figure 1a,b). The best discriminating accuracy of the autonomic indices was 0.71% for the sympathetic index and 0.69% for the parasympathetic index (Table 3).

### 3.2. Machine Learning 

#### 3.2.1. Feature Importance and Feature Selection

**Figure 2:**
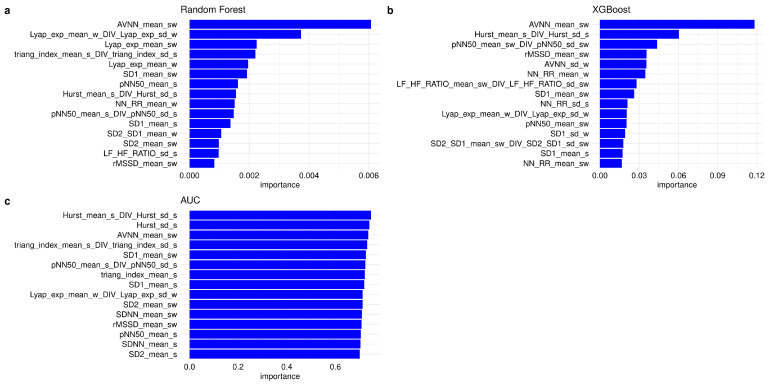
Feature importance evaluated by ML algorithms: (**a**) Random Forest model, (**b**) XGBoost model, and (**c**) ROC-AUC.

Random Forest, XGBoost, and ROC-AUC algorithms were used to evaluate the feature importance, as shown in Figure 2a–c. The normalized Hurst coefficient during sleep (H_norm_s), defined as the ratio between the mean and the standard deviation of the Hurst coefficient evaluated on sleep segments, showed the best discriminating power using ROC AUC importance, and its performances in classifying iRBD subjects with and without PLMS using a Logistic Regression model with LOO-CV were as follows: accuracy, 0.71 (0.55–0.84); AUC, 0.71; sensitivity, 0.70; specificity, 0.74; PPV, 0.76; NPV, 0.67. The eight most important features used in the SVM model, selected according to ROC-AUC importance, were as follows: the normalized Hurst coefficient, the standard deviation of the sleep Hurst coefficient, the sleep-to-wake ratio of mean AVNN, the normalized sleep triangular index, the sleep-to-wake ratio of mean SD1, the normalized sleep pNN50, the mean sleep triangular index, and the mean sleep SD1. The twelve most important features according to the Random Forest algorithm were as follows: the sleep-to-wake ratio of the mean AVNN, the normalized wake Lyapunov exponent, the sleep-to-wake ratio of the Lyapunov exponent, the normalized sleep triangular index, the mean wake Lyapunov exponent, the sleep-to-wake ratio of SD1, the mean sleep pNN50, the normalized sleep Hurst coefficient, the mean wake NN-to-RR ratio, the normalized sleep pNN50, the mean sleep SD1, and the mean wake SD2-to-SD1 ratio. XGBoost identified the sleep-to-wake ratio of the mean AVNN, the normalized sleep Hurst coefficient, the normalized sleep-to-wake ratio of the mean pNN50, the sleep-to-wake ratio of the mean rMSSD, the standard deviation of the wake AVNN, the mean wake NN-to-RR ratio, and the normalized sleep-to-wake ratio of the LF/HF ratio as the seven features with the highest importance. The subsets of the most important features identified by the ROC-AUC, RF, and XGBoost did not fully overlap. However, all the ML algorithms confirmed that the normalized sleep Hurst coefficient was one of the most important features selected. Sex and age did not contribute to the predictions, as their calculated importance in discriminating RBD with PLMS from those without PLMS was very low. 

#### 3.2.2. Classification Performance of ML Models in Distinguishing iRBD with and without PLMS

**Figure 3:**
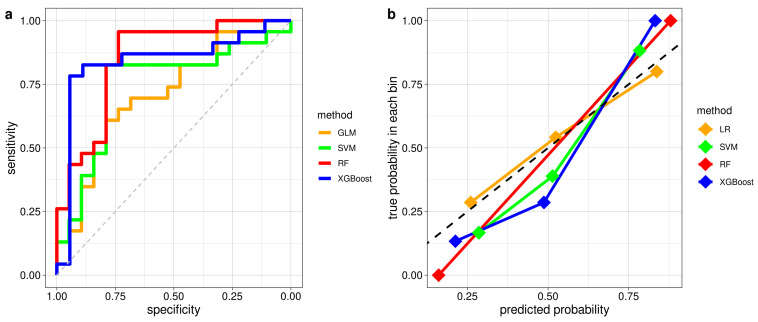
ROC curves (**a**) and calibration plots (**b**) from LOO-CV of optimal models with the best hyperparameters tuning: Logistic Regression (AUC = 0.71), Support Vector Machine (AUC = 0.75), Random Forest (AUC = 0.76), and XGBoost (AUC = 0.84). ROC: Receiver Operating Characteristic; LOO-CV: Leave-one-Out Cross Validation; AUC: Area Under the Curve.

Figure 3 shows the ROC and calibration curves of the optimal ML models trained with LOO-CV for their best performing sets of features. The hyperparameters of the ML models, obtained from the tuning process, are listed in Table S1 (Supplementary Material). Table 3 reports the classification accuracies (with their 95% confidence interval), AUCs, sensitivities, specificities, PPVs, and NPVs for each ML model. The RF model trained using its twelve most important features achieved the best classification accuracy of 0.86. The sensitivity, specificity, PPV, and NPV were 0.96, 0.74, 0.81, and 0.93, respectively. The classification accuracy was a bit higher than the AUC corresponding to this optimal model (AUC = 0.85). The SVM model, trained on its eight most important features, reached a classification accuracy of 0.81, with a sensitivity of 0.83, specificity of 0.79, ppv of 0.83, and npv of 0.79. The XGBoost model, trained on its seven most important features, achieved a 0.78 classification accuracy and AUC of 0.84, with a sensitivity of 0.83, specificity of 0.72, ppv of 0.79, and npv of 0.76. A comparison between the classification accuracies of ML models and cardiac autonomic indices is also reported in Table 3.

## 4. Discussion

In this study, we investigated the performance of ML models in differentiating iRBD patients with PLMS from those without PLMS. Our study demonstrates that ML algorithms using a combination of HRV parameters can accurately differentiate between these two iRBD phenotypes. In detail, while the RF model performed the best with an 86% accuracy, SVM achieved an accuracy of 81%, and the XGBoost algorithm showed a LOO-CV accuracy of 78%. In contrast, LR, cardiac sympathetic index, and cardiac parasympathetic index did not perform with acceptable classification performances in differentiating between these two iRBD groups, if compared to the other ML models.

In our study, we also compared the demographic and clinical features of iRBD patients with and without PLMS. No between-group differences were detected, and the two groups were clinically indistinguishable. Indeed, only slight differences in age, age at onset of iRBD symptoms, and education level were detected. These findings are in accordance with previous evidence investigating the clinical significance of PLMS in iRBD, reporting similar clinical characteristics for both phenotypes [31]. On the other hand, our iRBD-PLMS patients showed a greater number of synucleinopathy phenoconversion biomarkers, in particular hyposmia and deficits in attentive and visuo-spatial domains, than those with iRBD. Cognitive dysfunctions have been widely described in patients with iRBD, especially in those with neurodegenerative disorders including DLB [4]. Conversely, patients with DLB exhibit PLMS with a higher index value than that found in patients with Alzheimer's Disease [32]. However, it remains to be elucidated whether iRBD converters in DLB also showed PLMS and whether DLB with PLMS also presented RBD. This last issue could likely be that RBD is a symptom in the core diagnostic criteria for DLB [33]. Taken together, these findings indicate that distinguishing patients with iRBD associated with PLMS from those without remains challenging. This question is of interest since it still remains to be determined whether PLMS modifies the rate and/or risk of phenoconversion of IRBD to neurodegenerative disorders and, for this reason, it may be considered as a biomarker.

This is the first study to investigate the cardiac autonomic changes in iRBD patients with and without PLMS using HRV analysis. Interestingly, during 24 h recordings, iRBD-PLMS patients showed an increase in cardiac autonomic indices compared to those without PLMS. Dynamic changes in the autonomic nervous system activity have been widely observed during nocturnal sleep in other sleep disorders associated with PLMS such as RLS and PLM disorder (PLMD) [34]. In PLMD, the PLMS-related cardiac sympathetic activation during the sleep stage has been detected several seconds before the occurrence of PLMS. In contrast, an increase in cardiac parasympathetic activity (index calculated as the ratio of HF components during night/day) has been reported in the group with PLMS. We believe that this discrepancy is due to two factors. Firstly, different disorders are correlated with different PLMS pathogenesis. Secondly, there are different modalities of investigation, such as long versus short recording. Supporting this, it has been demonstrated that an initial activation of sympathetic activity occurs in iRBD patients, later followed by a more prolonged bradycardia [5]. Finally, the classification performances of the cardiac autonomic indices for differentiating iRBD with and without PLMS have been also calculated. Both the cardiac sympathetic and parasympathetic indices showed a low accuracy of 71% and 69%, respectively. These findings are not surprising, since similar results have been previously published regarding the differentiation between iRBD and controls [13]. Overall, our results suggest that HRV parameters should be used with caution because of the possibility of overlap between the iRBD groups.

In the last few years, (AI) models have gained growing capabilities in assisting clinicians with the diagnosis of neurological disorders and in supporting the differential diagnosis between clinically indistinguishable phenotypes [34,35]. Interestingly, in this study, we present novel ML models built on HRV parameters for distinguishing iRBD patients with and without PLMS. In detail, we used the RF model trained on the twelve most important features (sleep-to-wake ratio of the mean AVNN, the normalized wake Lyapunov exponent, the sleep-to-wake ratio of the Lyapunov exponent, the normalized sleep triangular index, the mean wake Lyapunov exponent, the sleep-to-wake ratio of SD1, the mean sleep pNN50, the normalized sleep Hurst coefficient, the mean wake NN-to-RR ratio, the normalized sleep pNN50, the mean sleep SD1, and the mean wake SD2-to-SD1 ratio) to differentiate iRBD patients with and without PLMS. According to our findings, the RF model showed both high classification accuracy (86%) and AUC (85%), whereas the SVM with the linear kernel model achieved a cross-validated accuracy of 81% (AUC = 75%). The classification performance of the XGBoost model trained on the seven most important features (sleep-to-wake ratio of mean AVNN, the normalized sleep Hurst coefficient, the normalized sleep-to-wake ratio of mean pNN50, the sleep-to-wake ratio of the mean rMSSD, the standard deviation of the wake AVNN, the mean wake NN-to-RR ratio, and the normalized sleep-to-wake ratio of the LF/HF ratio) was also tested. The XGBoost model showed a lower accuracy (equal to 78%) than the RF and SVM models, probably due to its inclusion in the model of the LF/HF ratio features. However, the AUC corresponding to this optimal model was equal to 84%. Moreover, the sensitivity of the ML models was different, with XGBoost lower (83%) than the RF model (96%). Dataset imbalance may be an important problem, as pointed out in [36,37]. However, in our study, the imbalance was not actually an issue. Classes were represented with a minority-to-majority ratio of 0.45 to 0.55. There was a slight imbalance that did actually does not affect performances.

To the best of our knowledge, only one antecedent study applied ML algorithms using HRV features in iRBD patients without PLMS, with excellent accuracy in differentiating patients from controls [13]. The current study represents a further step forward in identifying iRBD with PLMS. A new method for the automatic detection of PLMS has been previously proposed for other sleep disorders; however, it requires electromyographic recording [38]. Finally, our ML models have been trained on the HRV parameters acquired from electrocardiographic recordings. Considering that the difference between electrocardiographic and photoplethysmography signals is small [39], in the future, we will investigate ML models trained on RR intervals obtained from pulse photoplethysmography.

This study, however, has some limitations. First, the sample size of our cohort was small. PLMS, however, are often clinically misdiagnosed, requiring polysomnographic confirmation for a correct diagnosis. Therefore, the results presented in this study need to be validated using a larger sample of iRBD subjects with and without PLMS to confirm the usefulness of ML models in clinical practice. Second, as our ML models were evaluated using only a sample extracted from an Italian population, further testing should be performed in other ethnic and geographical groups. Third, studies evaluating patients from other sleep centers are needed in order to ensure the generalizability of our findings. However, this study has clear strengths. First, our proposed ML models accurately identified iRBD patients with PLMS. Second, AI algorithms were trained using HRV features only, which, beyond being very easy to obtain, are non-invasive measures of particular practical value for differentiating among clinically indistinguishable phenotypes. Finally, although our results are preliminary and based on a small sample size, we found that iRBD patients with PLMS showed a greater number of synucleinopathy biomarkers than those without PLMS. Longitudinal studies are needed to determine whether these iRBD patients convert faster to defined neurodegenerative disorders over time. In conclusion, our results demonstrate that ML models based on features derived from HRV analysis are useful for identifying iRBD patients with and without PLMS. The implementation of AI tools in a software application could support clinicians in properly diagnosing iRBD patients with or without PLMS.

## Figures and Tables

**Table 1:** Comparisons of demographic, clinical, and electrophysiological features of patients affected by iRBD with and without PLMS.

Variables	Total iRBD Group (*N* = 42)	iRBD-PLMS (*N* = 23)	iRBD (*N* = 19)	*p*-Value
**Demographics**				
Sex: No. men/women ^#^	31/11	16/7	15/4	0.74
Age, years (mean ± SD) ^$^	69.52 ± 7.90	71.26 ± 5.85	67.42 ± 9.59	0.29
Education level, years (mean ± SD) ^$^	11.64 ± 4.00	11.04 ± 4.24	12.37 ± 3.67	0.21
**Disease features**				
Disease duration, years (mean ± SD) ^$^	4.05 ± 3.30	4.57 ± 3.78	3.42 ± 2.57	0.33
Age at onset of iRBD, years (mean ± SD) ^$^	65.69 ± 8.30	66.61 ± 7.42	64.58 ± 9.34	0.65
RBD symptoms on Video-PSG recording, *n* (%)				
-Motor Agitation and Vocalization ^#^	16 (38.1)	9 (39.1)	7 (36.9)	1
-Motor Agitation ^#^	9 (21.4)	4 (17.4)	5 (26.3)	0.75
-Vocalization ^#^	4 (9.5)	2 (8.7)	2 (10.5)	1
-PSG features without clinical signs ^#^	13 (31)	8 (34.8)	5 (26.3)	0.80
PLMS index (mean ± SD) ^$^	27.47 ± 27.49	46.45 ± 23.66	4.49 ± 4.51	0
**Motor Evaluation**				
-Part III of UPDRS scale (mean ± SD) ^$^	1.44 ± 1.89	1.80 ± 2.30	1.00 ± 1.20	0.58
**Neuropsychological Battery**				
-MMSE (mean ± SD) ^$^	28.35 ± 1.42	28.51 ± 1.36	28.16 ± 1.51	0.35
-Token test (mean ± SD) ^$^	32.17 ± 2.13	32.55 ± 1.67	31.71 ± 2.56	0.14
-RAVLT D.R. (mean ± SD) ^&^	5.26 ± 0.76	5.27 ± 0.82	5.25 ± 0.70	0.96
-RAVLT I.R. (mean ± SD) ^&^	43.86 ± 7.71	44.90 ± 6.70	42.61 ± 8.80	0.36
-Raven’s Progressive Matrices (mean ± SD)	30.21 ± 3.19	30.35 ± 2.69	30.05 ± 3.77	0.78
-Corsi block-tapping Test (mean ± SD) ^&^	4.46 ± 0.90	4.53 ± 0.83	4.39 ± 0.99	0.63
-Digit Span Forward (mean ± SD) ^$^	5.77 ± 0.86	5.66 ± 0.85	5.90 ± 0.88	0.65
-Digit Span Backward (mean ± SD) ^&^	4.26 ± 0.89	4.53 ± 0.82	4.39 ± 0.98	0.39
-Verbal Fluency with Phonemic cues (mean ± SD) ^&^	32.71 ± 9.81	30.96 ± 7.97	34.84 ± 11.53	0.22
-Verbal Fluency with Semantic cues (mean ± SD) ^&^	45.98 ± 7.57	46.48 ± 7.29	45.37 ± 8.05	0.65
-Attentive Matrices (mean ± SD) ^$^	47.95 ± 6.38	48.43 ± 6.25	47.37 ± 6.65	0.98
-Copy Rey–Osterrieth complex figure (mean ± SD) ^&^	32.30 ± 4.26	32.60 ± 3.62	31.93 ± 5.02	0.82
**Cardiac Autonomic Evaluation**				
-Cardiac Sympathetic Index (mean ± SD) ^$^	3.47 ± 4.15	3.43 ± 3.13	3.51 ± 5.22	0.38
-Cardiac Parasympathetic Index (mean ± SD) ^$^	3.09 ± 3.15	3.95 ± 3.98	2.04 ± 1.06	0.19

IRBD: idiopathic REM sleep behavior disorder; PLMS: periodic leg movements during sleep; PSG: polysomnographic; MMSE: Mini-Mental State Examination; RAVLT I.R: Auditory–Verbal Learning Test Immediate; RAVLT-DR: Auditory–Verbal Learning Test Delayed; Italian Population Normal Values: MMSE: >23.80; Token test: >26.5; RAVLT I.R.: >28.53; RAVLT D.R.: >4.69; Raven’s Progressive Matrices: >18; Corsi block-tapping Test: >3.46; Digit Span Forward: >4.26; Digit Span Backward: >2.65; Verbal Fluency with Phonemic cues: >25; Verbal Fluency with Semantic cues: >17; Attentive Matrices: >31; Copy Rey–Osterrieth complex figure: >28.88. ^#^ Χ^2^ test; ^$^ Mann–Whitney U test; ^&^ Student’s *t*-test.

**Table 2:** Comparisons of synucleinopathies phenoconversion biomarkers in iRBD patients with and without PLMS.

Variables	iRBD-PLMS (*N* = 23)	iRBD (*N* = 19)	*p*-Value ^$^
**Phenoconversion Biomarkers, *n* (%)**			
– Hyposmia/Olfactory deficits	13 (56.5)	3 (15.7)	0.02
– Cognitive Impairment	11 (47.8)	4 (21.0)	0.14
– Abnormal 18F-FDG PET	10 (43.5)	2 (10.5)	0.04
– Motor Symptoms	4 (17.4)	2 (10.5)	0.85
**Patients with phenoconversion biomarkers, *n* (%)**			
– at least 1 biomarker	22 (95.6)	9 (47.3)	0.001
– 1 biomarker	10 (45.5)	7 (77.8)	0.90
– 2 biomarkers	9 (41)	2 (22.2)	0.08
– 3 biomarkers	2 (9)	-	0.56
– 4 biomarkers	1 (4.5)	-	1

iRBD: idiopathic REM sleep behavior disorder; PLMS: periodic leg movements during sleep; 8F-FDG PET imaging: Fluorodeoxyglucose PET. ^$^ Χ^2^ test.

**Table 3:** Classification performances of Machine Learning (ML) models and cardiac autonomic indices.

	Accuracy (95% conf. int.)	AUC	Sensitivity	Specificity	ppv	Npv
**ML Models**						
LR	0.71 (0.55–0.84)	0.71	0.70	0.74	0.76	0.67
SVM	0.81(0.66–0.91)	0.75	0.83	0.79	0.83	0.79
RF	0.86 (0.71–0.95)	0.85	0.96	0.74	0.81	0.93
XGBoost	0.78 (0.62–0.89)	0.84	0.83	0.72	0.79	0.76
**Autonomic Indices**						
Sympathetic	0.71 (0.60–0.83)	0.70	0.70	0.74	0.78	0.67
Parasympathetic	0.69 (0.55–0.81)	0.63	0.70	0.68	0.73	0.65

LR: Logistic Regression; SVM: Support Vector Machine; RF: Random Forest; XGBoost: eXtreme Gradient Boosting; AUC: Area Under the (ROC) Curve.

## Data Availability

The data presented in this study are available on request from the corresponding author. The data are not publicly available due to privacy restrictions.

## Supplementary Materials

The following supporting information can be downloaded at: https://www.mdpi.com/article/10.3390/diagnostics14040363/s1, Table S1: Hyperparameters tuning of Random Forest and eXtreme Gradient Boosting models.
