# Supplementary material for: Periodic Leg Movements during Sleep Associated with REM Sleep Behavior Disorder: A Machine Learning Study
Source: Diagnostics (Basel). 2024 Feb 7;14(4):363. doi: 10.3390/diagnostics14040363 (PMC10888394; doi:10.3390/diagnostics14040363)
Supplement: Supplementary file 1 [file diagnostics-14-00363-s001.zip › diagnostics-2771261-supplementary.pdf]

Supplementary Material

Table S1. Hyperparameters tuning of Random Forest and eXtreme Gradient Boosting models

Model	Hyperparameters	definition
SVM	C = 0.7368	Cost parameter
RF	Mtry = 1	Number of variables randomly sampled as candidates at each split
XGBoost	Nrounds = 400	Maximum number of iterations/trees to grow
	Max.depth = 3	Depth of the tree
	Eta = 0.1	Learning rate
	Gamma = 0.9	Regularisation (preventing overfitting)
	Col.sample_by_tree=0.4	Controls the number of features supplied to a tree
	Child_weight = 2	Leaf threshold for stopping tree splitting
	Subsample = 0.5	Controls the number of samples supplied to a tree

SVM: Support Vector Machine; RF: Random Forest; XGBoost: eXtreme Gradient Boosting.
